# Comparative Mechanisms of Graphene Nanoplatelets and Graphene Oxide in Regulating Pore Structure and Drying Shrinkage of Cement Mortar

**DOI:** 10.3390/ma19194253

**Published:** 2026-10-08

**Authors:** Junliang Zhao, Junjie He, Dongyan Wu, Zhongkun Wang, Gengying Li, Jingjing Huang

**Affiliations:** 1 College of Civil Engineering and Architecture, Wenzhou University, Wenzhou 325035, China; zhaojunliang@wzu.edu.cn (J.Z.); hjj1401452119@163.com (J.H.); 00151070@wzu.edu.cn (D.W.); 2 Key Laboratory of Engineering and Technology for Tideland Reclamation and Life-Cycle Intelligent Monitoring of Zhejiang Province, Wenzhou 325035, China; 3 School of Civil Engineering and Transportation, Foshan University, Foshan 528225, China; ligengying@fosu.edu.cn; 4 School of Civil Engineering & Architecture, Wenzhou Polytechnic University, Wenzhou 325035, China

**Keywords:** graphene, graphene oxide, cement mortar, nano-reinforcement, drying shrinkage, microstructure

## Abstract

**Highlights:**

- Systematic comparison of GNP vs. GO reinforcement in cement mortar.
- GO shows superior enhancement in strength, water absorption, and shrinkage control.
- Optimal dosages identified: 0.05% for flexural strength/shrinkage, 0.03% for compressive strength/water absorption.
- GO refines pore structure: 15.0% porosity reduction and 51.3% large-pore (>100 nm) reduction.

**Abstract:**

This study comparatively investigates the reinforcement mechanisms of graphene nanoplatelets (GNPs) and graphene oxide (GO) in cement mortar at dosages of 0–0.1%, focusing on mechanical properties, drying shrinkage, fluidity, water absorption, and microstructural evolution characterized by SEM, XRD, and MIP. Both nanomaterials reduce fluidity, with GO exhibiting a less pronounced effect. The mechanical properties display unimodal dosage-dependent trends, with optimal flexural strength at 0.05% and optimal compressive strength at 0.03%; the corresponding 28 d enhancements reach 16.9% and 20.8% for GO, compared with 8.2% and 8.5% for GNP. Water absorption and drying shrinkage attain minima at 0.03% and 0.05%, respectively. GO consistently outperforms GNP, which is attributed to its superior pore refinement, reducing total porosity by 15.0% and large pores (>100 nm) by 51.3%, owing to its functional groups that promote dispersion and hydration nucleation. These results establish GO as a more effective nano-reinforcement for pore-structure engineering in cementitious composites.

## 1. Introduction

As a typical porous and hydrophilic material, cement concrete contains a large number of fine pores and inherent microcracks. Changes in environmental humidity cause the migration and loss of pore water, inducing volume shrinkage and thereby triggering drying shrinkage cracks [1]. Such cracks not only directly impair the structural integrity and durability of concrete but also provide penetration pathways for aggressive agents such as carbon dioxide, chloride ions, and moisture. This significantly accelerates carbonation, freeze–thaw damage, and reinforcement corrosion, ultimately jeopardizing structural safety and service life. The essence of drying shrinkage deformation lies in the migration of physically adsorbed water from C–S–H gel and water from capillary pores within the cement paste. The extent of shrinkage is primarily governed by the amount and rate of evaporable water migration, both of which are directly controlled by the material’s internal pore structure and microcrack network. The higher the porosity and the more developed the crack network, the easier the water migration pathways and the more pronounced the drying shrinkage [2,3]. Therefore, improving the concrete’s microstructure, reducing harmful pores, inhibiting microcrack initiation and propagation, and reducing the migration rate of capillary water are key strategies for controlling drying shrinkage deformation.

In recent years, the introduction of nanomaterials has provided new approaches for enhancing the performance of cement mortar. Graphene (GNP), a two-dimensional crystalline material composed of single-layer carbon atoms with sp^2^ hybridization, exhibits exceptional mechanical strength, high specific surface area, and outstanding electrical and thermal conductivity [4]. Studies have shown that an appropriate amount of GNP can effectively improve the mechanical properties and durability of cementitious matrices [5,6,7]. Its derivative, graphene oxide (GO), is rich in hydrophilic oxygen-containing functional groups such as hydroxyl and carboxyl groups on its surface [8], endowing it with good dispersibility in water. Leveraging this characteristic, GO not only densifies the microstructure through physical filling within the cement matrix [9] but also forms chemical bonds with cement hydration products via its surface functional groups [10]. This physical–chemical synergistic effect provides an ideal pathway to enhance the mechanical properties, toughness, and durability of cement mortar. Regarding mechanical properties, Zhang et al. [11] found that incorporating 0.03% GO increased the 28-day flexural and compressive strength of cement mortar by 22% and 37%, respectively. Similar trends have also been observed by Matalkah et al. [12], Peng et al. [13], and other researchers [14,15,16,17]. In terms of durability, GO also enhances the resistance of cement mortar to sulfate attack, chloride ion penetration, and freeze–thaw cycles [18,19,20,21,22]. Furthermore, GO promotes cement hydration and refines the pore structure through a templating effect, while simultaneously bridging microcracks via its two-dimensional lamellar structure, thereby improving the macroscopic performance of cement mortar [23]. Although these studies have confirmed the reinforcing potential of GNP and GO, most of them focused on a single nanomaterial and employed different dispersion methods, dosages, and curing conditions. As a result, the reported optimal dosages and strengthening efficiencies vary considerably among studies, making it difficult to directly compare the relative effectiveness of GNP and GO.

However, while GO improves mechanical properties, it also has two major application bottlenecks. First, due to its extremely high specific surface area and surface activity, GO significantly reduces the fluidity of cement paste, adversely affecting workability. Second, and more critically, the strong van der Waals forces and π–π interactions between GO layers make them highly prone to agglomeration. Without uniform and stable dispersion, the reinforcing effect is greatly diminished, and may even negatively impact performance. To address the dispersion challenge of GO, a combination of chemical and physical methods is typically employed [24]. Chemical methods primarily utilize dispersants to isolate GO layers through steric hindrance or electrostatic repulsion, while physical methods rely on ultrasonic treatment, mechanical stirring, and other techniques to supply energy for exfoliation. To date, systematic investigations into the effects of different dispersion processes on the workability and mechanical properties of cement mortar remain limited.

From the perspective of dispersion methods, the effectiveness of chemical dispersants, ultrasonic treatment, and mechanical stirring depends strongly on the type, dosage, and surface chemistry of the nanomaterial [4,24]. However, a unified dispersion protocol has not yet been established, and the dispersion stability of GNP and GO under identical conditions has rarely been compared. Regarding pore refinement, both GNP and GO can refine the pore structure of cementitious materials, but the relationship between pore structure evolution and macroscopic shrinkage behavior remains insufficiently quantified [9,25]. With respect to hydration mechanisms, GO mainly promotes hydration through its oxygen-containing functional groups, whereas GNP acts primarily through physical nucleation and filling effects; nevertheless, direct comparative evidence under consistent conditions is still scarce. In terms of shrinkage control, existing studies have mostly examined either GNP or GO alone, and the role of capillary pore refinement in the 5–50 nm range has not been systematically linked to the Kelvin and Young–Laplace capillary tension mechanisms [26].

Therefore, this study conducts a systematic comparison of GNP and GO in cement mortar under identical dispersion, mixing, and curing conditions. The novelty lies in three aspects. First, GNP and GO are compared at the same dosage levels using the same dispersion process, so differences in their reinforcing mechanisms can be attributed to their intrinsic structural and chemical characteristics rather than to experimental conditions. Second, a comprehensive comparative framework is established, covering fluidity, mechanical properties, water absorption, drying shrinkage, and microstructure. Third, the drying shrinkage behavior is interpreted by linking the capillary pore refinement observed in the MIP data with the Kelvin and Young–Laplace capillary tension mechanisms. These findings are expected to clarify the relative advantages of GNP and GO and to support the rational design of nano-reinforced cementitious composites.

## 2. Materials and Methods

### 2.1. Materials

The materials included P.O 42.5 ordinary Portland cement (Wenzhou Conch Cement Co., Ltd.; Wenzhou, China, see Table 1), standard sand (Xiamen ISO Standard Sand Co., Ltd., Xiamen, China), graphene (GNP) and graphene oxide (GO) (Suzhou Tanfeng Graphene Technology Co., Ltd., Xiamen, China; parameters in Table 2), and Sika polycarboxylate superplasticizer (Shanghai Chenqi Chemical Technology Co., Ltd., Shanghai, China).

A total of 11 mix proportions were designed in this experiment. The effects of graphene (GNP) at mass fractions of 0%, 0.01%, 0.03%, 0.05%, 0.075%, and 0.1% and graphene oxide (GO) at mass fractions of 0%, 0.01%, 0.03%, 0.05%, 0.075%, and 0.1% were investigated. These mixes are designated as GNP1–GNP5 and GO1–GO5, respectively, with PCC as the control. The specific mix proportions are shown in Table 3.

### 2.2. Experimental Methods

(1) Dispersion method: First, measure the required amount of tap water into a beaker. Add the weighed polycarboxylate powder to the beaker and stir until evenly mixed. After it has completely dissolved, add the weighed graphene/graphene oxide to the beaker and stir until evenly mixed. Subsequently, place the beaker on a magnetic stirrer and stir for 30 min. After stirring, transfer the beaker to an ultrasonic cleaner and sonicate for 30 min to obtain the corresponding graphene/graphene oxide solution.

(2) Fluidity Test Method: The fluidity of the cement-based material was determined according to GB/T 2419-2005 [27]. Before material preparation, the flow table surface, mold inner walls, tamper, and other tools were wiped with a wrung-out wet cotton cloth, and the mold was centered on the flow table. After mixing, the mold was held steady, and the mortar was quickly loaded into the truncated conical mold in two layers. The first layer, filled to about 2/3 of the mold height, was scored five times in two perpendicular directions and uniformly tamped 15 times. The second layer, filled to approximately 20 mm above the mold rim, was tamped similarly. Following tamping, the collar was removed, and excess mortar flush with the mold top was scraped off twice using a knife held at an angle, followed by wiping away any mortar fallen onto the table. The truncated conical mold was then slowly and vertically lifted, and the flow table was immediately started to perform 25 jolts within 25 s ± 1 s at 1 Hz. After jolting, the spread diameters in two perpendicular directions were measured with an electronic digital caliper.

(3) Flexural Strength Test Method: Prismatic specimens (40 mm × 40 mm × 160 mm) were used for the flexural strength test. After preparation, they were placed in a standard curing room until reaching the specified age. Upon removal, surface water was wiped off, and the test was performed following GB/T 17671-2021 [28] using a YAW-300D fully automatic pressure testing machine. For each curing age and mix proportion, three specimens were tested at a loading rate of 50 N/s, with the failure load automatically recorded by the instrument. Before testing, the testing machine was connected to the power supply and debugged to meet the requirements. The appearance of each specimen was inspected, and those with obvious defects were discarded. The specimens were placed one by one into the flexural test fixture, ensuring that each specimen was centered in the testing machine. After specimen failure, the failure load was recorded. The flexural strength was calculated according to Equation (1).

(1) $$R_{f}=\frac{1.5F_{f}L}{bh^{2}}$$

where $R_{f}$—flexural strength (MPa); $F_{f}$—failure load (N); *L*—distance between support points (100 mm); *b*—width of the prism cross-section (40 mm); *h*—height of the prism cross-section (40 mm).

(4) Compressive Strength Test Method: The compressive test specimens were the half-prism specimens obtained after the flexural strength test, with a compressive surface dimension of 40 mm × 40 mm. Each prism specimen yielded two test pieces after fracture, resulting in a total of six compressive test specimens per group. The compressive test was conducted immediately after the flexural strength test. Before testing, any moisture and debris on the specimen surface were wiped clean to ensure a flat compressive surface. The loading rate was 2.4 kN/s, and the maximum load at specimen failure was automatically controlled and recorded by the testing machine. All specimens were tested and the data were recorded individually. The compressive strength of the small cube was calculated according to Equation (2).

(2) $$R_{c}=\frac{F_{c}}{A}$$

where $R_{c}$—compressive strength (MPa); $F_{c}$—maximum failure load (N); *A*—area of the compressive surface (mm^2^).

(5) Water Absorption Test Method: The water absorption test was conducted according to JGJ/T 70-2009 [29]. After curing to the specified age, specimens were removed, surface-dried, and then oven-dried at (80 ± 5) °C for (48 ± 2) h, after which the absolute dry mass (m_0_) was recorded. Each specimen was then placed with its molded surface facing downward and fully submerged in a water tank, keeping the top surface at least 20 mm below the water surface. Following (24 ± 0.5) h of immersion, the specimens were taken out, surface water blotted with a wrung-out wet cloth, and the wet mass (m_1_) recorded. Water absorption was calculated using Equation (3), and the arithmetic mean of three specimens was taken, accurate to 1%.

(3) $$W_{x}=\frac{m_{1}-m_{0}}{m_{0}}\times 100\%$$

where $W_{x}$—water absorption of the mortar (%); $m_{0}$—mass of the dry specimen (g); $m_{1}$—mass of the specimen after water absorption (g).

(6) Drying Shrinkage Test Method: The test followed JGJ/T 70-2009 [29]. Prismatic specimens of 40 mm × 40 mm × 160 mm were used. The fresh cement-based mixture was cast into molds and cured at (20 ± 2) °C with >90% relative humidity for 24 h, then demolded. Both ends of each specimen were marked to indicate the test direction, and copper pins were affixed to the center of each end face using 502 adhesive. After 4 h for adhesive curing, the initial length was measured along the marked direction with an electronic digital caliper. The specimens were then placed in a controlled environment at (20 ± 2) °C and (60 ± 5)% RH. Length readings (as dried lengths) were taken at 1, 3, 7, 14, 28, 60, 90, and 180 days. The drying shrinkage value was then calculated using Equation (4).

(4) $$\varepsilon_{t}=\frac{l_{0}-l_{t}}{l}$$

where $\varepsilon_{t}$—natural drying shrinkage value of the mortar specimen at t days; $l_{0}$—length of the specimen after molding (including measuring pins), i.e., the initial length (mm); $l_{t}$—measured length of the specimen at t days (including measuring pins) (mm); l—reference length of the specimen (mm).

The final drying shrinkage value, accurate to 1.0 × 10^−4^, represents the arithmetic mean of three specimens.

(7) Microscopic testing: The microscopic testing experiments were conducted by sending the samples to a testing agency. For SEM observation, three samples with a size of about 1 cm × 1 cm × 1 cm were prepared for each mix at the age of 28 days from failed specimens after the strength test. The SEM tests were performed using a Zeiss Gemini 300 (Carl Zeiss AG, Oberkochen, Germany). For MIP analysis, samples of approximately 3–5 mm were taken from the core of the specimens at 28 days, and dried under vacuum before testing. The MIP tests were carried out using an AutoPore IV 9510 (Micromeritics Instrument Corp., Norcross, GA, USA). The XRD analysis was conducted using an Ultima IV instrument (Rigaku Corp., Tokyo, Japan) on powder samples ground from the 28-day specimens.

## 3. Results

### 3.1. Effect of Graphene on the Fluidity of Cement Mortar

**Figure 1:**
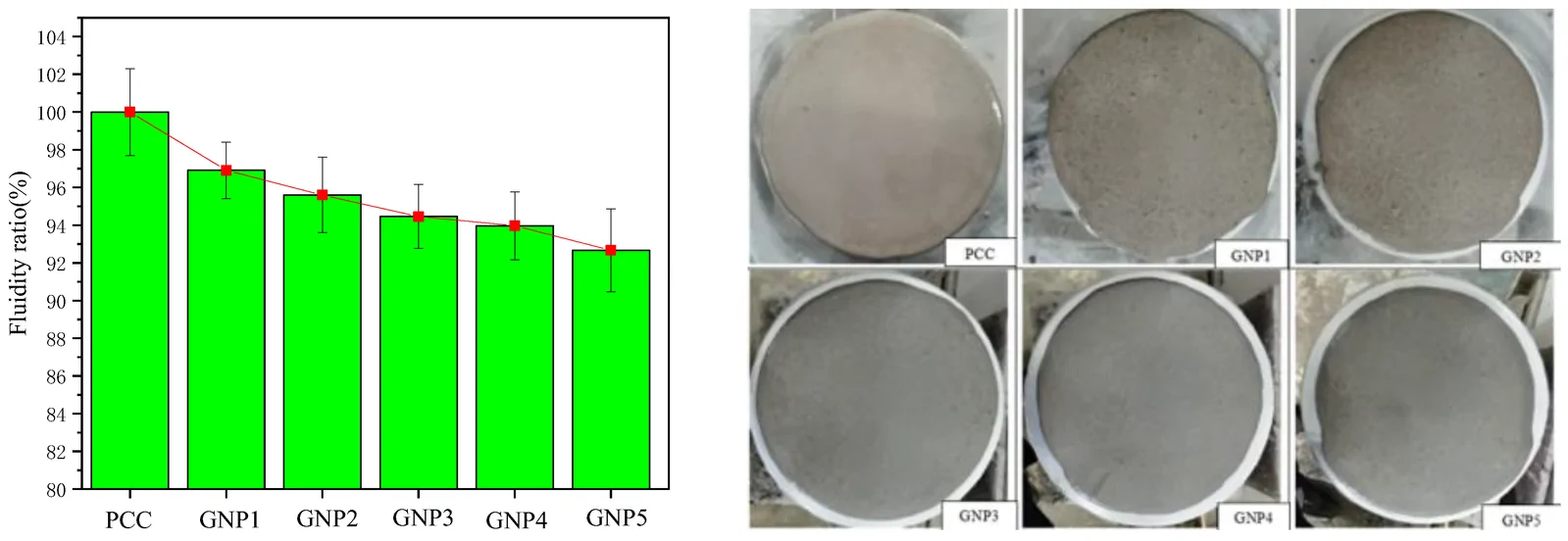
Effect of GNP content on flow properties of mortar.

The effect of GNP incorporation on mortar fluidity is shown in Figure 1. It can be observed that as the GNP content increases, the fluidity of the mortar gradually decreases. When the GNP content increased from GNP1 to GNP5, the fluidity decreased by 3.1%, 4.4%, 5.5%, 6.0%, and 7.3%, respectively, compared to the PCC group. This indicates that the introduction of GNP adversely affects the rheological properties of the composite material. A possible explanation is that GNP, due to its strong hydrophobicity, large specific surface area, and significant van der Waals forces and π–π stacking interactions between layers, is highly prone to agglomeration [30]. The agglomerated GNP adheres to the surfaces of cement particles, increasing the internal friction within the paste and leading to a rise in plastic viscosity, which may contribute to the gradual decrease in fluidity with increasing GNP content.

### 3.2. Effect of Graphene Oxide on the Fluidity of Cement Mortar

**Figure 2:**
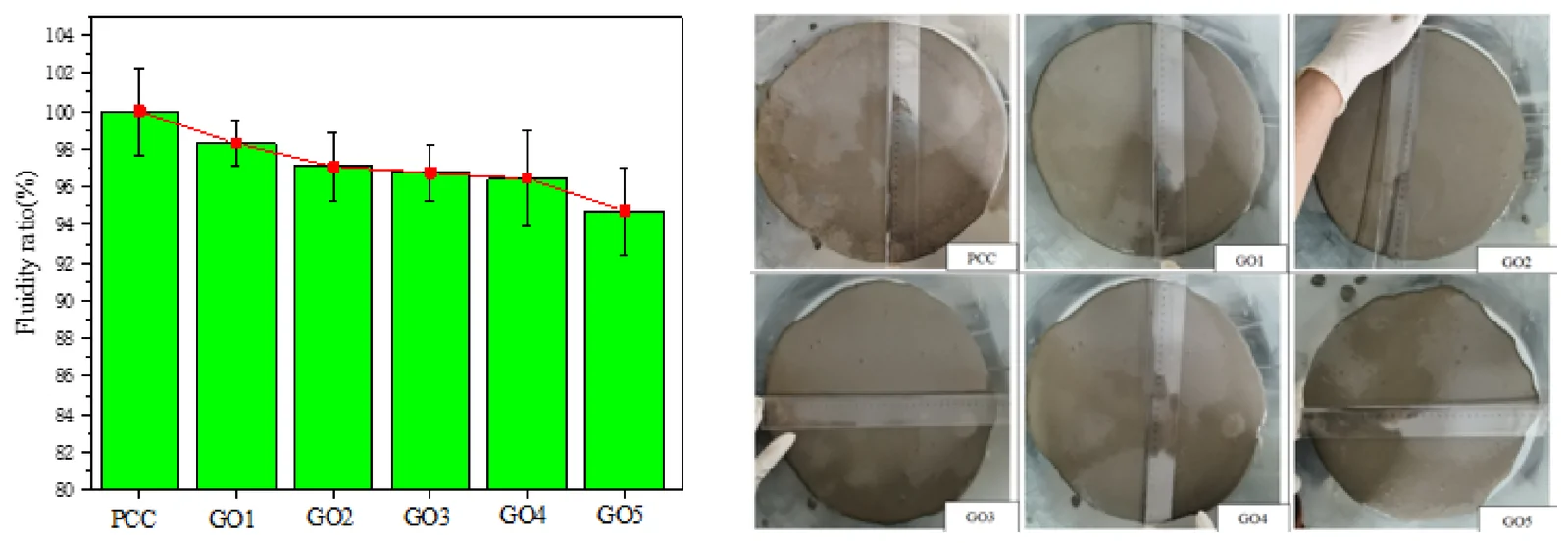
Effect of GO dosage on the fluidity of mortar.

The effect of GO incorporation on mortar fluidity is shown in Figure 2. As the GO content increases, the fluidity of the mortar gradually decreases. For GO1–GO5, the fluidity decreased by 1.7%, 2.9%, 3.3%, 3.6%, and 5.3%, respectively, compared to the control group. The incorporation of GO also impairs the fluidity of the mortar, but its negative impact is significantly less pronounced than that of the GNP-incorporated system (at the same dosage of 0.1%, GO reduces fluidity by 5.3%, while GNP reduces it by 7.3%). This difference in mechanism stems from the unique structure of GO: GO possesses a two-dimensional lamellar structure with abundant oxygen-containing functional groups such as hydroxyl and carboxyl groups on its surface and edges, endowing it with strong hydrophilicity. These groups bind to water molecules through hydrogen bonding, adsorbing and immobilizing part of the mixing water, thereby possibly reducing the lubricating effect of free water and leading to decreased fluidity [31,32]. Although GO also reduces fluidity, the reduction is more moderate, which is mainly attributed to the better dispersibility of GO, reducing the additional frictional resistance caused by severe agglomeration as observed with GNP.

### 3.3. Effect of Graphene on the Mechanical Properties of Cement Mortar

**Figure 3:**
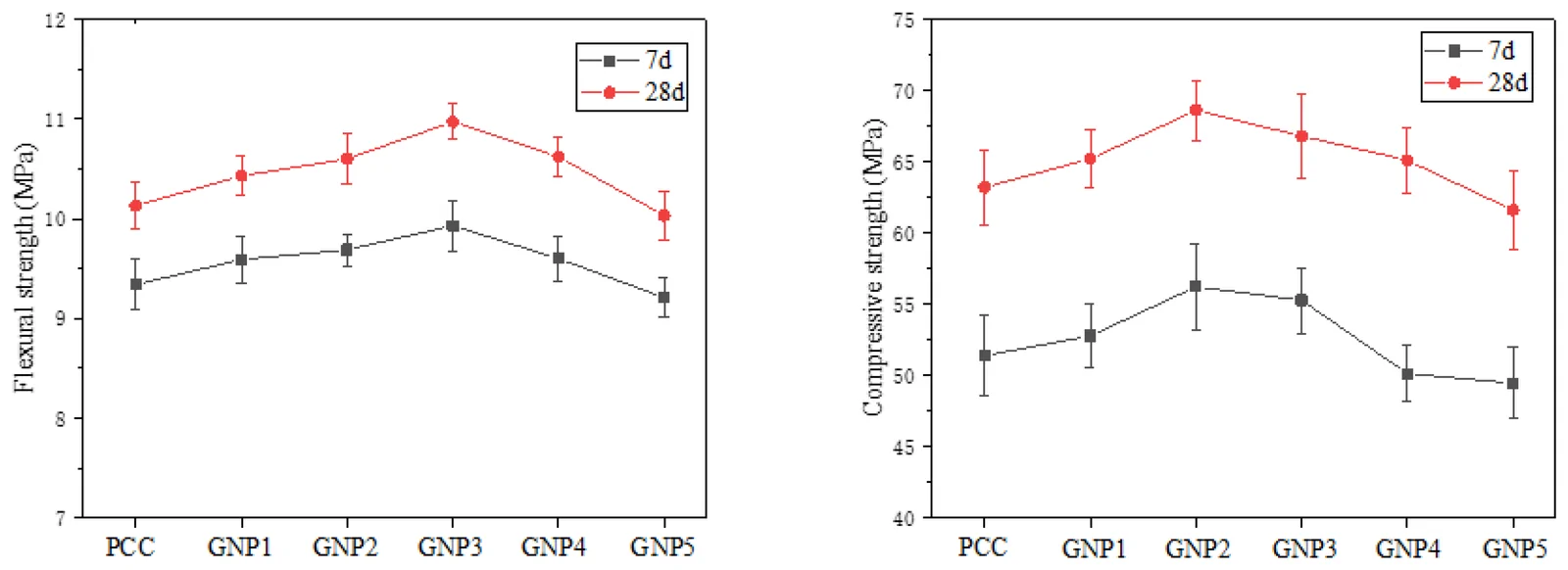
Flexural and compressive strength of GNP-modified mortar at 7 days and 28 days.

The effect of GNP on the flexural and compressive strength of mortar is shown in Figure 3. It can be observed that the mechanical properties of GNP-modified cement mortar exhibit a typical dose-dependent characteristic. At the age of 7 days, the flexural strength reached its peak at a GNP content of 0.05% (GNP3), showing an increase of 6.2% compared to the PCC group; the compressive strength peaked at a content of 0.03% (GNP2), showing an increase of 9.4% compared to the PCC group. When the content increased to 0.1% (GNP5), the flexural strength decreased by 1.4% compared to the PCC group. At the age of 28 days, GNP3 increased the flexural strength by 8.2%, reaching 11.0 MPa (compared to 10.2 MPa for the PCC group); GNP2 increased the compressive strength by 8.5%, reaching 68.6 MPa (compared to 63.2 MPa for the PCC group). At a content of 0.1% (GNP5), the flexural and compressive strengths decreased by 1.4% and 3.8%, respectively, compared to the PCC group. This indicates that an appropriate amount of GNP can effectively enhance the mechanical properties of the specimens, but excessive incorporation leads to adverse effects.

Within the dosage range of 0–0.1%, the effect of GNP incorporation on the mechanical properties of cement mortar follows a pattern of initial enhancement followed by reduction. When incorporated in an appropriate amount, the nanoscale size and ultra-high specific surface area of GNP enable it to act as nucleation sites for hydration products, promoting uniform growth of C-S-H gel. Meanwhile, GNP can fill micropores in the matrix, increasing compactness, thereby improving both flexural and compressive strength. However, when the content is too high, GNP tends to agglomerate due to strong interlayer van der Waals forces and π–π stacking interactions, making uniform dispersion difficult. These agglomerates not only hinder the development of hydration reaction interfaces but also disrupt the continuity of the C-S-H gel due to their large size and inert surfaces, forming pores and weak interfaces around the agglomerates, ultimately leading to a decline in mechanical properties.

### 3.4. Effect of Graphene Oxide on the Mechanical Properties of Cement Mortar

**Figure 4:**
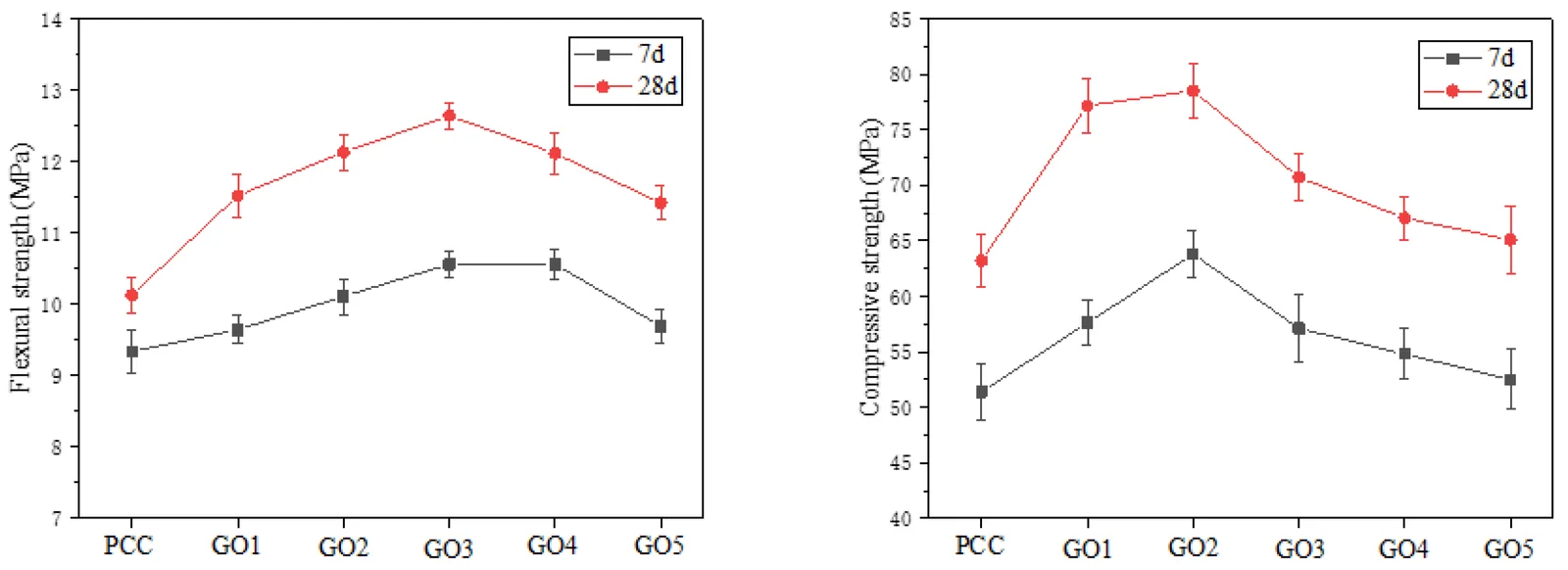
Flexural and compressive strength of GO-modified mortar at 7 days and 28 days.

As shown in Figure 4, GO improves the mechanical properties of cement-based composites at low dosages but reduces them at high dosages. At the age of 7 days, the flexural strength reached its peak at a GO content of 0.05% (GO3), showing an increase of 12.1% compared to the PCC group; the compressive strength showed the maximum increase of 20.7% at a content of 0.03% (GO2). When the content increased to 0.1% (GO5), performance degradation occurred, with the compressive strength decreasing by 0.54%. At the curing age of 28 days, GO3 increased the flexural strength by 16.9%, reaching 12.6 MPa; GO2 increased the compressive strength by 20.8%, reaching 78.5 MPa. Compared to the GNP-only system, GO exhibited a greater strengthening efficiency at the age of 28 days: the compressive strength increase rate was 12.3% higher, and the flexural strength increase rate was 8.7% higher.

When an appropriate amount of GO is incorporated, the abundant oxygen-containing functional groups on its surface may provide nucleation sites for cement hydration products, accelerating the hydration reaction and promoting the formation of C-S-H gel [25,33,34]. Meanwhile, GO exerts a templating effect, guiding hydration products to form a dense clustered structure, and tightly connects the hydration product crystals through nanoscale effects and bridging action, potentially reducing internal microcracks and pores within the matrix, thereby enhancing both flexural and compressive strength. However, when the GO content exceeds the critical value of 0.05%, negative effects gradually emerge: on the one hand, GO agglomerates cause localized stress concentrations within the cement paste, weakening structural integrity; on the other hand, excessive incorporation significantly reduces the fluidity of the paste, affecting bubble release and leading to increased internal porosity. This indicates that the agglomeration effect of GO gradually dominates when the dosage exceeds the critical value, leading to the appearance of an inflection point in mechanical properties.

### 3.5. Effect of Graphene on the Water Absorption of Cement Mortar

**Figure 5:**
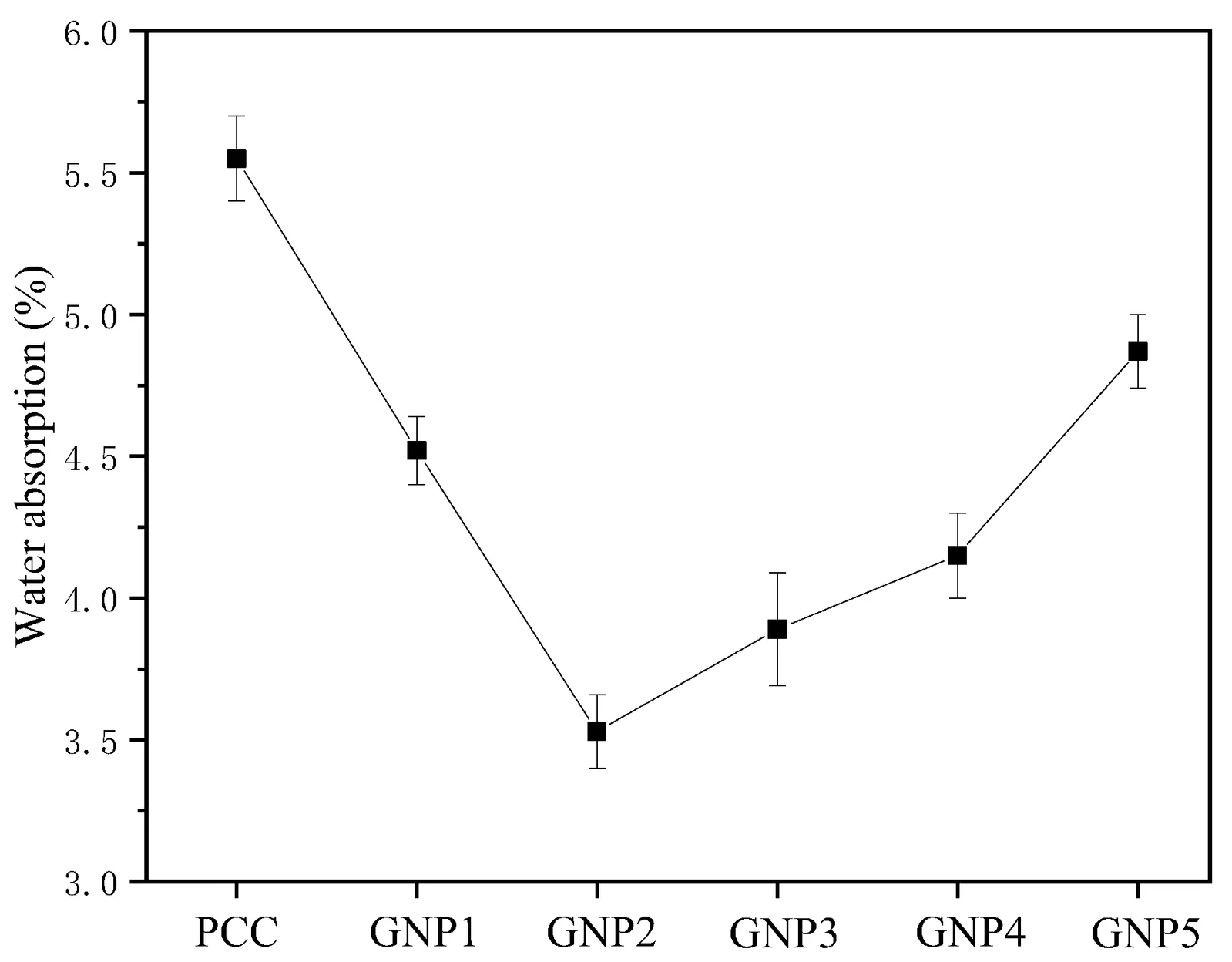
Effect of GNP dosage on water absorption of mortar.

The water absorption results of graphene-modified cement-based composites are shown in Figure 5. The water absorption exhibits a trend of first decreasing and then increasing with the increase in graphene content. The water absorption reached its lowest level at a graphene content of 0.03% (GNP2), representing a 35.2% reduction compared to the ordinary cement-based material group. All groups incorporating graphene showed reduced water absorption relative to the ordinary cement-based material group, with GNP1, GNP3, GNP4, and GNP5 exhibiting reductions of 17.2%, 28.3%, 23.1%, and 12.8%, respectively. This is because, at a low dosage, graphene, as a nanoscale material, can effectively fill cracks, voids, and other defects generated during the hydration process within the cement matrix, increasing the internal compactness of the cement-based material and reducing water ingress through pores, thereby lowering water absorption. However, as the graphene content further increases, due to the poor dispersibility of graphene in water, excessive graphene sheets aggregate and stack to form “graphene mountains.” The rough surface of the stacked graphene sheets also hinders the growth of C-S-H gel, affecting the cement hydration process and leading to the reappearance of pores, cracks, and other defects within the cement matrix. Consequently, the solution can easily penetrate the cement matrix through these defects, causing an increase in water absorption.

### 3.6. Effect of Graphene Oxide on the Water Absorption of Cement Mortar

**Figure 6:**
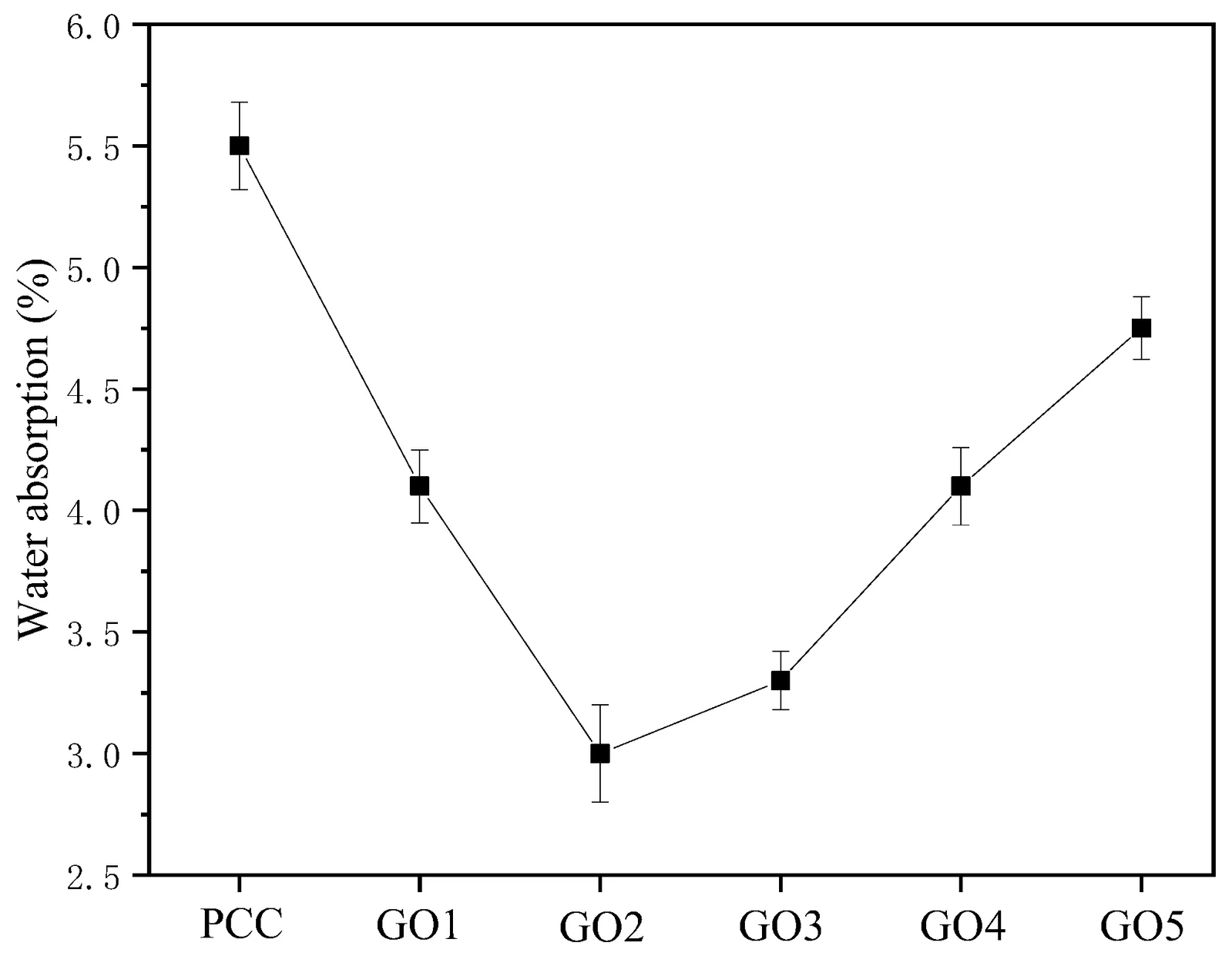
Effect of GO dosage on water absorption of mortar.

The water absorption results of graphene oxide-modified cement-based composites are shown in Figure 6, which illustrates the variation in water absorption of GO1–GO5. It can be observed that the water absorption of the cement-based material exhibits a trend of first decreasing and then increasing with the increase in graphene oxide content. The water absorption reached its lowest level at GO2, representing a 49.5% reduction compared to the ordinary cement-based material group. It can also be observed that all test groups incorporating graphene oxide showed reduced water absorption compared to the non-incorporated group across different curing ages, with GO1, GO3, GO4, and GO5 exhibiting reductions of 30.8%, 44.6%, 30.9%, and 19.8%, respectively, relative to the ordinary cement-based material group. This is because the two-dimensional flake structure of a small amount of graphene oxide can fill micropores, cracks, and gaps between unhydrated cement particles within the cement-based material, reducing pore connectivity and thereby decreasing water penetration pathways. The oxygen-containing functional groups (such as carboxyl and hydroxyl groups) on the surface of graphene oxide can form chemical bonds or physical adsorption with cement hydration products (such as C-S-H gel), enhancing interfacial bonding and reducing interfacial defects, further inhibiting water migration. Consequently, the incorporation of graphene oxide leads to a reduction in water absorption. However, as the graphene oxide content further increases, agglomeration occurs, and its two-dimensional flake structure may form localized agglomerates due to excessive stacking, creating new “weak zones” or large pores between cement particles. These defects become rapid channels for water penetration, conversely increasing water absorption. Moreover, compared to the groups incorporating only graphene, the groups incorporating only graphene oxide exhibit a better effect in reducing water absorption.

### 3.7. Effect of Graphene on the Drying Shrinkage Performance of Mortar

**Figure 7:**
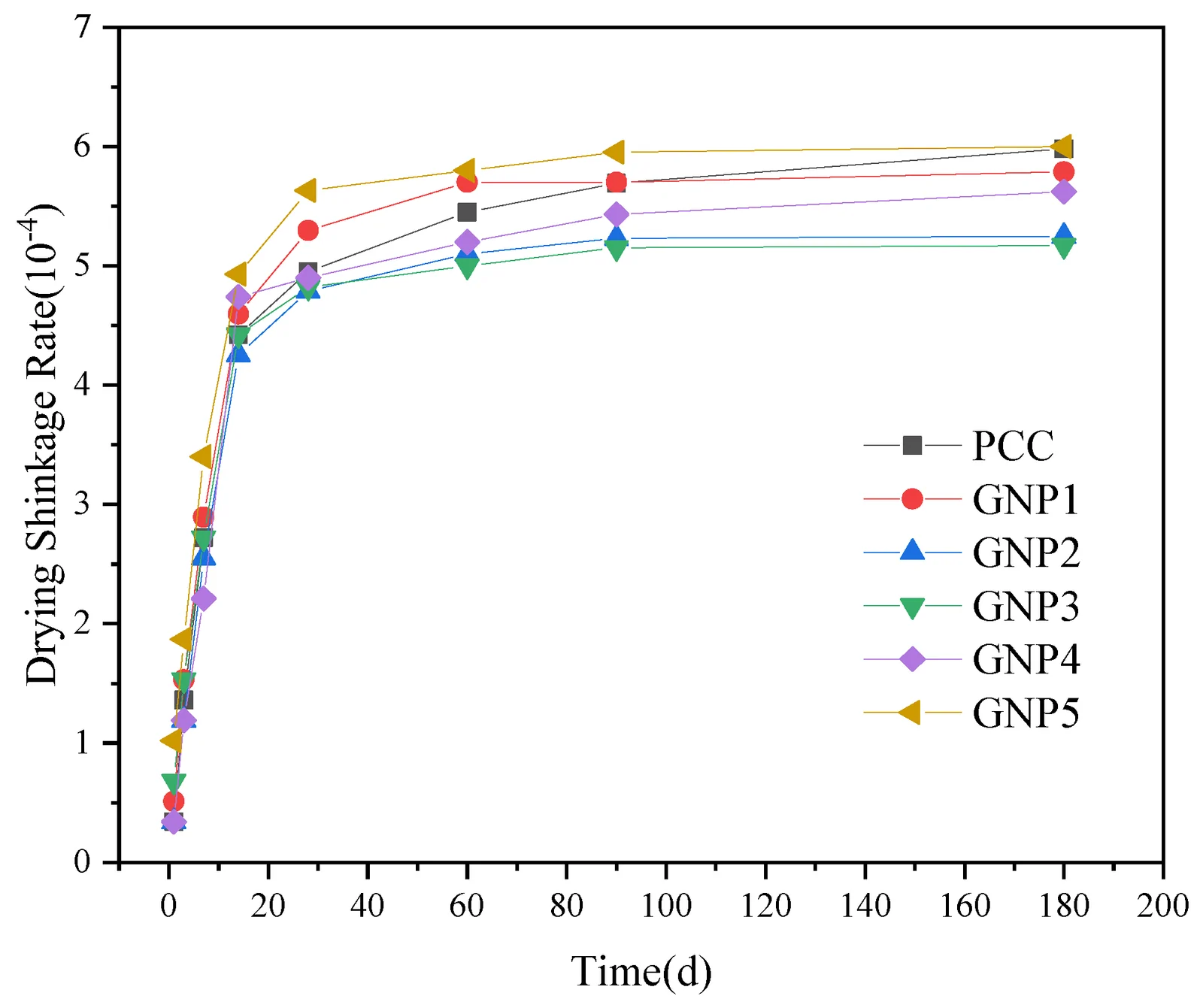
Drying shrinkage of GNP-modified mortar.

Figure 7 illustrates the evolution of drying shrinkage of cement mortar with curing age under different GNP dosages. All specimens exhibited a rapid increase in shrinkage within the early stage (within 28 days), followed by a gradual stabilization in the later stage (after 90 days). At a low GNP dosage (GNP1), the drying shrinkage of the mortar increased by 6.9% compared to the non-incorporated group at 28 days, but the difference gradually narrowed with age, ultimately showing a 3.3% reduction at 180 days, generally comparable to the non-incorporated group. At a high dosage (GNP5), the drying shrinkage at all ages was higher than that of the non-incorporated group, increasing by 13.7% and 0.3% at 28 days and 180 days, respectively; the increase was more significant in the early stage, but with increasing age, the drying shrinkage gradually approached that of the non-incorporated group, and the two were essentially consistent at 180 days. When the GNP dosage ranged from GNP2 to GNP4, the drying shrinkage was lower than that of the non-incorporated group: it remained roughly unchanged or slightly decreased within 28 days, and the reduction became significant after 28 days. Specifically, at GNP2, the drying shrinkage at 60, 90, and 180 days decreased by 6.4%, 8.1%, and 12.4%, respectively; at GNP3, the decreases were 8.3%, 9.5%, and 13.5%, respectively. The reduction in drying shrinkage helps inhibit the initiation and propagation of microcracks, which corroborates the aforementioned flexural and compressive strength test results, indicating that an appropriate amount of GNP incorporation can not only enhance the mechanical properties of cement mortar but also effectively reduce its drying shrinkage deformation.

### 3.8. Effect of Graphene Oxide on the Drying Shrinkage Performance of Mortar

**Figure 8:**
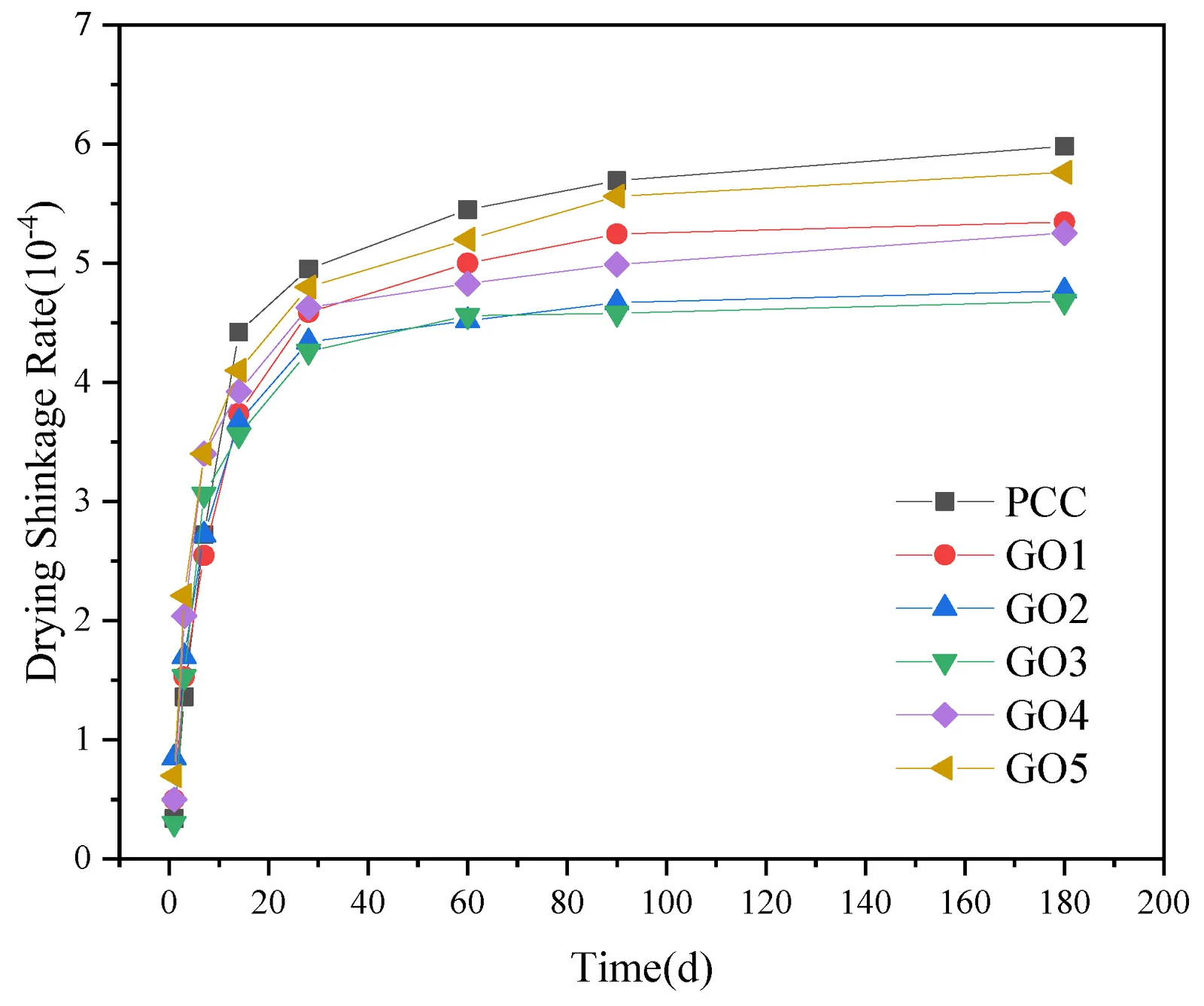
Drying shrinkage of GO-modified mortar.

Figure 8 illustrates the evolution of drying shrinkage of cement mortar with curing age under different graphene oxide dosages. Overall, the drying shrinkage of all specimen groups first increased rapidly with age and then gradually stabilized. Among them, the drying shrinkage of the non-incorporated mortar group continued to increase slowly in the later stage, with a growth of 20.8% at 180 days compared to that at 28 days. In contrast, for mortars incorporating GO2, GO3, and GO4, the drying shrinkage at 180 days increased by only 9.9%, 7.7%, and 9.1%, respectively, compared to that at 28 days. At low (GO1) or high (GO5) dosages, the drying shrinkage increases from 28 days to 180 days were 16.5% and 20.0%, respectively, which are close to the level of the non-incorporated group (20.8%). Compared to the control group, all mortars incorporating GO exhibited reduced drying shrinkage, which decreased first and then increased with increasing dosage. At GO3, the drying shrinkage reached its minimum at all ages: at 28, 60, 90, and 180 days, the drying shrinkage of this group decreased by 13.9%, 16.3%, 19.5%, and 21.7%, respectively, compared to the non-incorporated group. At the age of 180 days, the groups GO1, GO2, GO4, and GO5 exhibited drying shrinkage reductions of 10.6%, 20.3%, 15.5%, and 3.7%, respectively, compared to the non-incorporated group.

Comparing Figure 7 and Figure 8, GO exhibits a better inhibitory effect on mortar drying shrinkage than GNP, but both follow the same trend with varying dosage, i.e., the drying shrinkage first decreases and then increases with increasing dosage, reaching a minimum at a dosage of 0.05%. Within the dosage range of 0.01–0.1%, the drying shrinkage of GO-modified mortar at 180 days is lower than that of GNP-modified mortar, with reductions of 7.6%, 9.1%, 9.5%, 10.1%, and 4.0%, respectively, indicating that GO has a greater advantage than GNP in improving the drying shrinkage performance of cement mortar.

### 3.9. Microstructure

**Figure 9:**
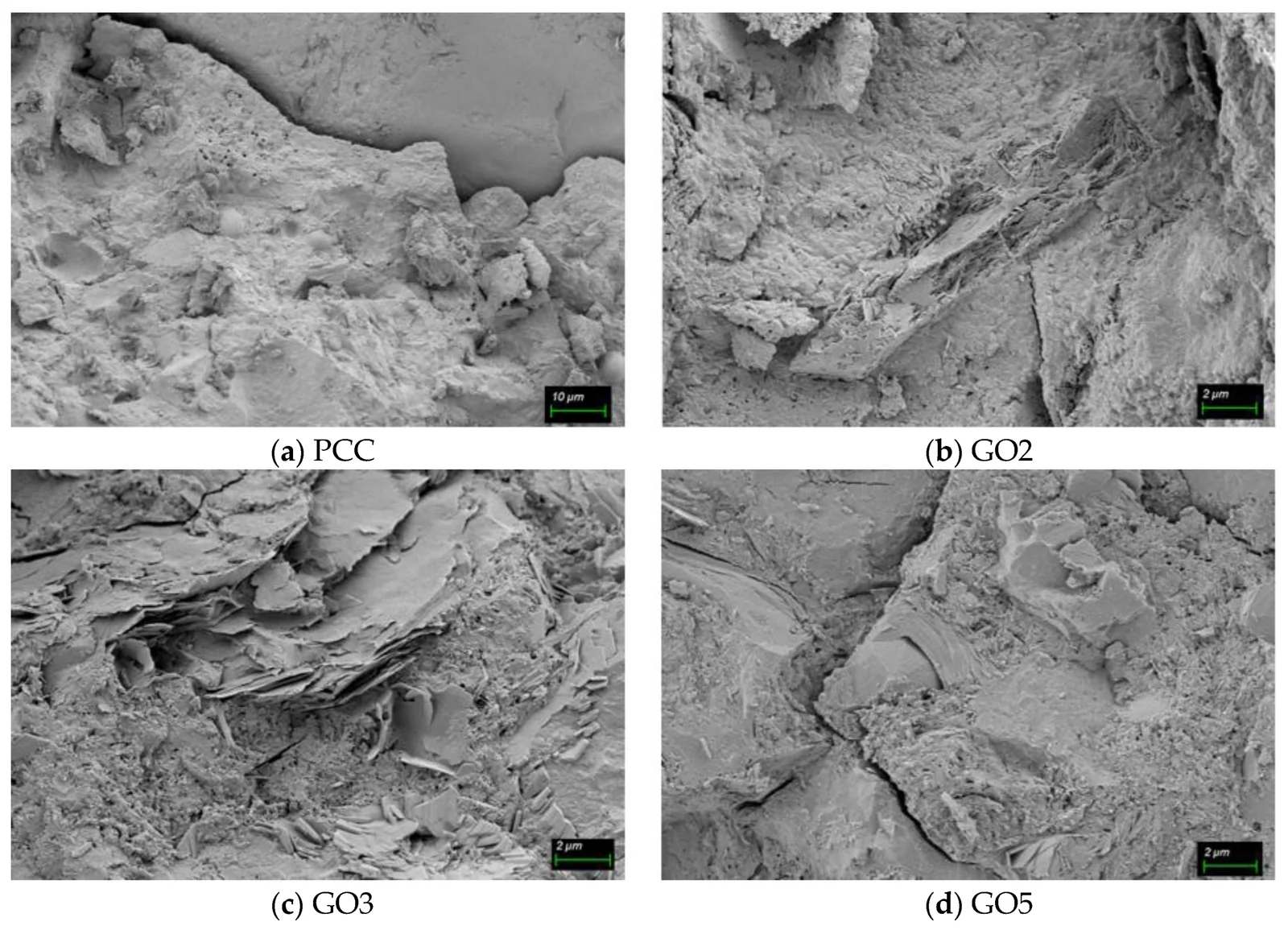
SEM images of PCC and GO-modified mortars: (**a**) PCC, (**b**) GO2, (**c**) GO3, (**d**) GO5.

The microstructure of graphene oxide-modified mortar is shown in Figure 9. The microstructure of ordinary cement-based material without GO incorporation is shown in Figure 9a, which exhibits a loose and porous characteristic, with hydration products distributed in a disordered manner, and visible pores and microcracks on the matrix surface, reflecting insufficient compactness due to inadequate hydration. The microstructure of GO2 is shown in Figure 9b, displaying a significantly improved morphology. Through the templating effect of GO, the hydration products form a dense clustered structure, with crystals interweaving to form a continuous network, and no obvious pores or cracks are observed in the matrix. This morphological feature confirms the mechanism by which GO promotes the directed growth of C-S-H gel by providing nucleation sites. The microstructure of GO3 is shown in Figure 9c. The paste still maintains relatively high compactness, but slight stacking of GO sheets begins to appear in local areas. Despite the presence of a small number of agglomerates, the clustered arrangement of hydration products remains dominant, and no new pores or microcracks emerge in the paste, indicating that the reinforcing effect of GO still outweighs the negative impact of agglomeration at this dosage. The microstructure of GO5 is shown in Figure 9d, where obvious structural deterioration is observed in the matrix. Excessive GO agglomerates form interlayer stacks, disrupting the continuity of hydration products, and microcracks and pore bands are visible in the matrix. This morphological feature is consistent with the declining trend in mechanical properties, confirming that the failure of GO dispersion due to excessively high dosage counteracts its microstructural regulation advantages.

The above morphological evolution pattern indicates that when an appropriate amount of GO is incorporated, its surface functional groups can guide the ordered growth of hydration products; however, when the dosage is too high, the agglomerates instead become internal defect sources. It is worth noting that the local stacking phenomenon observed in the 0.05% dosage system suggests that the dispersion stability of GO has already begun to decline at this critical optimal dosage, providing direct microstructural evidence for determining the optimal dosage range of GO.

**Figure 10:**
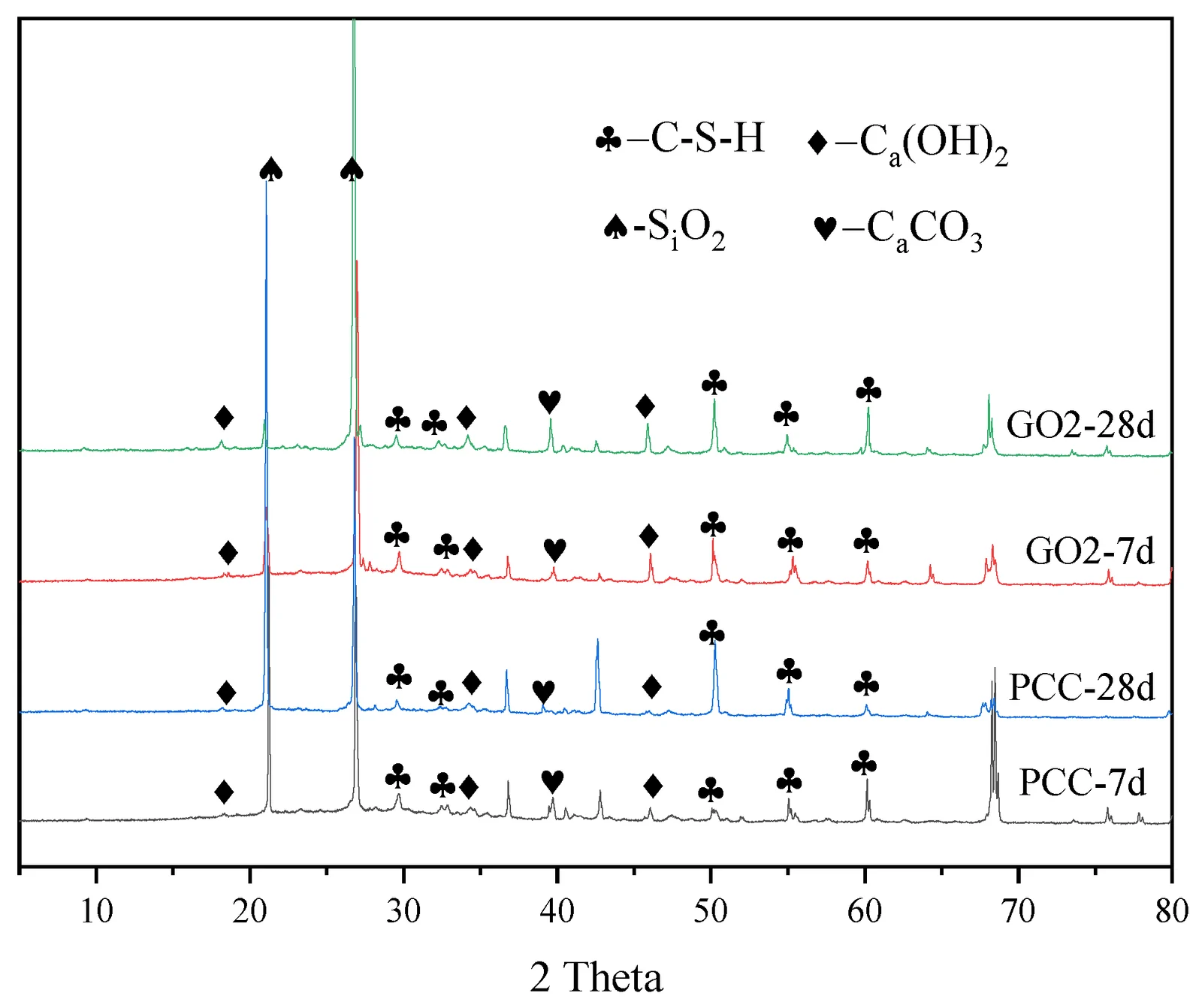
XRD patterns of PCC and GO2 at curing ages of 7 and 28 days.

Figure 10 presents the X-ray diffraction (XRD) patterns of the PCC and GO2 groups at curing ages of 7 and 28 days, providing an important basis for analyzing the composition and quantity of hydration products. Characteristic peaks of calcium silicate hydrate (C-S-H) appear at 2θ angles of 7°, 16°, 29°, 32°, 49°, 55°, and 60° [35,36]. Compared with the plain mortar, the mortar incorporating GO2 exhibits significantly increased peak intensities at 29° and 49°, indicating that GO promotes the cement hydration process and favors the formation of more calcium silicate hydrate. The peaks appearing at 2θ angles of 18.0°, 34.1°, and 47.1° correspond to calcium hydroxide (Ca(OH)_2_) [37,38]. It can be observed that the calcium hydroxide peak intensities of the GO2 group are also markedly higher than those of the plain group, especially the peak at 47.1°, which is significantly greater than that of the PCC group, indicating a more complete hydration process and the generation of more hydration products. A comparison of the patterns at 7 d and 28 d reveals that the peak intensities of the hydration products at 28 d are all greater than those at 7 d, further demonstrating that with increasing curing age, the hydration process becomes more complete and more hydration products are formed.

Table 4 presents the porosity results measured by mercury intrusion porosimetry (MIP) for four groups of mortar specimens (PCC, GO2, GO3, and GO5) after 28 days of curing. It should be noted that the MIP analysis was performed only on PCC and selected GO-modified mortars; the GNP-modified mortars were not characterized by MIP in this study. Therefore, the pore structure comparison in this section is limited to PCC and the selected GO mixtures. An appropriate dosage of graphene oxide significantly influenced the porosity and pore size distribution of the cement mortar. Compared with the PCC group, the total porosity of the GO2 group decreased by 15.0%, and the proportion of large pores (pore diameter > 100 nm) decreased by 51.3%; the total porosity and large pore proportion of the GO3 group decreased by 14.1% and 48.2%, respectively. The average pore diameter of the PCC group was 33.64 nm, while those of the GO2 and GO3 groups decreased to 24.88 nm and 26.54 nm, respectively, indicating a significant improvement in matrix compactness. However, in GO5, both the porosity and the proportion of large pores were higher than those of the PCC group, primarily because excessive GO agglomerated and could not disperse uniformly, leading to an increased number of large-diameter pores and higher porosity.

**Figure 11:**
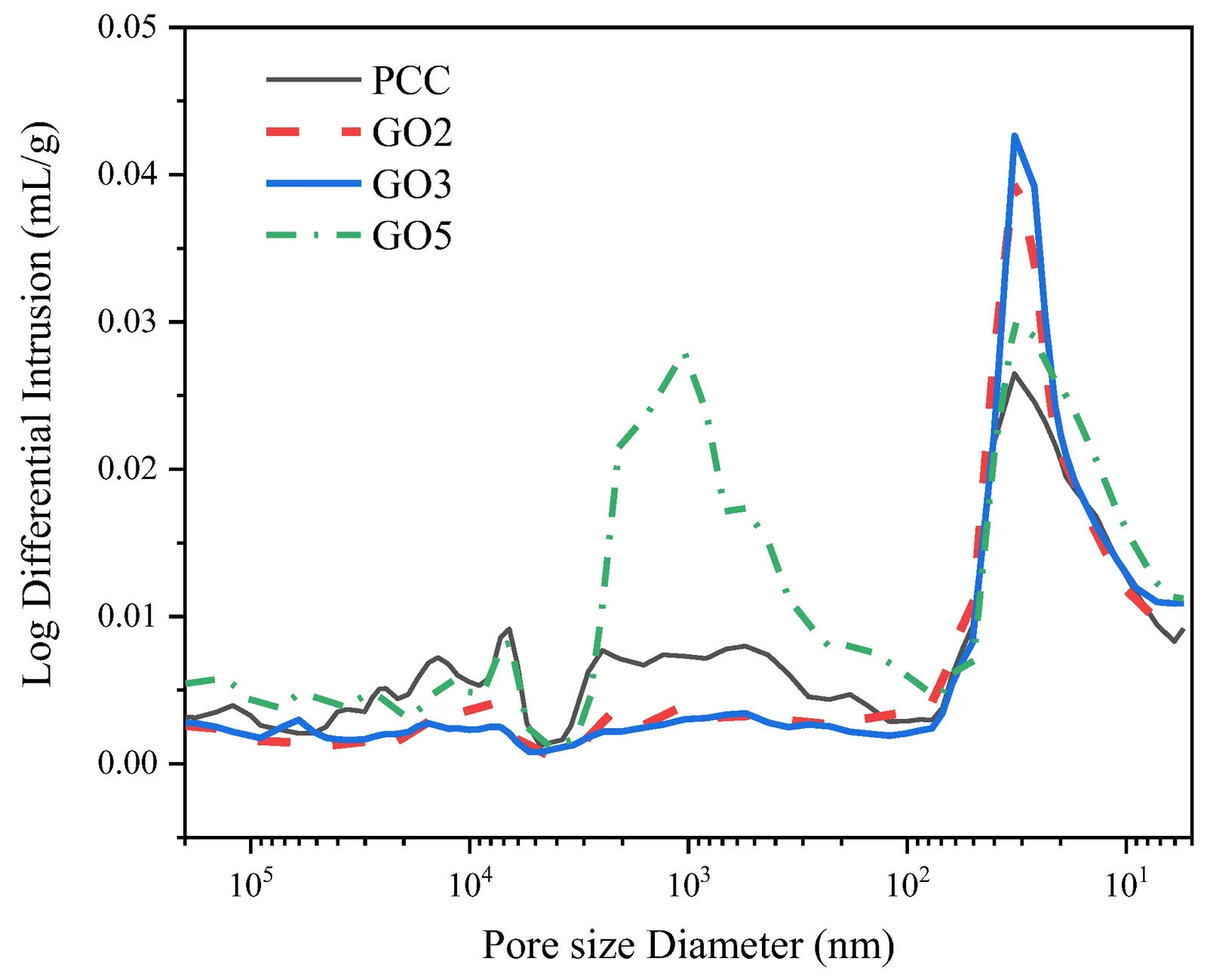
Pore size distribution of PCC, GO2, GO3, and GO5 mortars.

Figure 11 shows the variation in the logarithmic differential mercury intrusion volume with pore diameter. The PCC group curve exhibits multiple peaks at pore diameters of approximately 6000 nm, 2200 nm, 500 nm, and 30 nm; the GO5 group curve also shows three peaks at approximately 6000 nm, 800 nm, and 29 nm, with a relatively high peak at 800 nm, indicating a large proportion of large pore volume. In contrast, both the GO2 and GO3 group curves exhibit only a single peak (at approximately 30 nm), with no significant peaks in the pore diameter range above 100 nm, demonstrating that an appropriate amount of GO effectively optimizes the pore size distribution of the mortar.

## 4. Discussion

An appropriate amount of GNP can effectively regulate the early-age drying shrinkage behavior of cement mortar. Under suitable dosage conditions, GNP improves matrix compactness through nanoscale bridging and pore filling effects. When microcracks initiate, GNP can inhibit crack propagation and delay defect development. However, as drying shrinkage stress continues to accumulate, cracks undergo branched propagation along interfaces, resulting in the later-stage drying shrinkage at high dosages converging with that of the control group. Furthermore, when GNP is excessively incorporated, its agglomeration leads to stacking within the cement paste, creating internal defects and increasing drying shrinkage.

GO exhibits a significant improvement effect on the drying shrinkage of cement mortar, with its mechanism being fundamentally different from that of GNP. At an appropriate GO dosage, it not only enhances matrix compactness through filling effects but, more importantly, exerts a microstructural regulation function. On one hand, the hydrophilic nature of GO’s active groups enables it to stably adsorb moisture, delaying evaporation; on the other hand, its lamellar structure provides a growth template for hydration products, promoting the development of crystals into dense clustered morphologies, accelerating hydration reactions, and generating more hydration products [26]. This dual action effectively reduces porosity and inhibits water loss. Additionally, GO sheets can block moisture migration pathways, further reducing water evaporation and thereby lowering the drying shrinkage of the cement matrix. However, when the dosage exceeds the critical value of 0.05% (GO3), the tendency for sheet stacking leads to agglomeration, which instead creates localized pore defects within the matrix, weakening the material’s optimization effect. This stands in sharp contrast to the mechanism of GNP, which primarily relies on physical filling and bridging effects.

The drying shrinkage behavior can be further interpreted through the Kelvin and Young–Laplace capillary tension mechanisms. According to the Kelvin equation, the equilibrium vapor pressure above a curved meniscus decreases with decreasing pore radius, so water in smaller capillary pores evaporates at a later stage. The Young–Laplace equation further shows that capillary tension is inversely proportional to pore radius, making the 5–50 nm range the most critical interval for drying shrinkage. The MIP results show that an appropriate GO dosage (GO2 and GO3) reduces the total porosity and the proportion of large pores (>100 nm) while shifting the dominant pore size toward 5–50 nm. Although pores in this range are more sensitive to capillary tension, the reduced total pore volume, the decreased large-pore proportion, and the denser C–S–H network together limit both the amount and the rate of evaporable water migration. Consequently, the capillary tension is more uniformly distributed and the overall shrinkage is reduced, consistent with the minimum drying shrinkage observed at GO3 (0.05%). In contrast, the weaker pore refinement in the GNP-modified mortars leads to a less pronounced reduction in drying shrinkage, further supporting the proposed capillary-tension-controlled mechanism.

## 5. Conclusions

This study systematically evaluated the effects of graphene (GNP) and graphene oxide (GO) on the fluidity, mechanical properties, water absorption, drying shrinkage, and microstructure of cement mortar. The key findings are as follows:

1. Both GNP and GO reduce mortar fluidity, with GO exhibiting a smaller negative effect. As the dosage increases, fluidity decreases progressively for both materials. At GNP5/GO5, GNP reduces fluidity by 7.3%, while GO reduces it by only 5.3%.
2. Appropriate amounts of GNP or GO significantly enhance the flexural and compressive strength of mortar. For both materials, the strength first increases and then decreases with increasing dosage, and the reinforcing effect becomes more pronounced with age. For flexural strength: GNP3 increases the 7 d/28 d strength by 6.2%/8.2%, while GO3 increases it by 12.1%/16.9%. For compressive strength: GNP2 increases it by 9.4%/8.5%, while GO2 increases it by 20.7%/20.8%. The reinforcing effect of GO is approximately 2.2–2.4 times that of GNP. Excessive incorporation leads to strength degradation: the strength of GNP begins to fall below that of PCC at GNP4, whereas GO exhibits only slight degradation at GO5.
3. Appropriate amounts of both GNP and GO reduce mortar water absorption, following the same trend: as the dosage increases, water absorption first decreases and then increases, with the optimal dosage being GNP2/GO2. At this dosage, GNP and GO reduce water absorption by 35.2% and 49.5%, respectively, compared to the non-incorporated group, with the latter showing a more significant reduction.
4. The drying shrinkage of all specimens increases with age, with rapid growth in the early stage (before 28 days) followed by a gradual stabilization. Both GNP and GO exhibit the same trend in affecting drying shrinkage: as the dosage increases, drying shrinkage first decreases and then increases, with the optimal dosage being GNP3/GO3. At this dosage, the GNP group shows reductions in drying shrinkage at 60 d/90 d/180 d of 8.3%/9.5%/13.5%, respectively, compared to the non-incorporated group; the GO group shows more significant reductions of 16.3%, 19.5% and 21.7%, indicating that GO exhibits a superior inhibitory effect on drying shrinkage.
5. An appropriate amount of GO promotes the interweaving and interconnection of hydration crystals, forming a continuous network structure with no obvious pores or cracks in the matrix. In contrast, excessive GO leads to agglomeration, forming interlayer stacks that disrupt the continuity of hydration products, resulting in microcracks and pore bands in the matrix. Compared with PCC, the incorporation of GO2 reduces the total porosity of the mortar by 15.0% and the proportion of large pores (pore diameter > 100 nm) by 51.3%. The average pore diameter of PCC is 33.64 nm, while that of GO2 is refined to 24.88 nm, indicating a significant improvement in matrix compactness.

## Figures and Tables

**Table 1:** Physical and chemical properties of cement.

SiO_2_	Al_2_O_3_	CaO	MgO	Fe_2_O_3_	K_2_O	MnO	SO_2_	LOI	Specific Surface, Blaine, m^2^/kg	Compressive Strength, 28-Day, MPa
18.3	4.5	62.4	2.1	0.3	3.5	5.3	2.97	0.34	350	51.2

**Table 2:** Basic parameters of GNP and GO.

	Thickness (nm)	Lateral Size (μm)	Specific Surface Area (m^2^/g)	Carbon Content (%)	Oxygen Content (%)	Sulfur Content (%)
GO	1	0.2–10	50–150	<48	>42	<2
GNP	3.4–8	5–50	100–300	>95	0.5	0.5

**Table 3:** Mix proportion of GO- and GNP-modified mortar.

	w/c	Water	Cement	Sand	Superplasticizer	GNP	GO
		kg·m^−3^					
PCC	0.3	225	750	1500	7.5	0	0
GNP 1	0.3	225	750	1500	7.5	0.075	0
GNP 2	0.3	225	750	1500	7.5	0.225	0
GNP 3	0.3	225	750	1500	7.5	0.375	0
GNP 4	0.3	225	750	1500	7.5	0.5625	0
GNP 5	0.3	225	750	1500	7.5	0.75	0
GO1	0.3	225	750	1500	7.5	0	0.075
GO2	0.3	225	750	1500	7.5	0	0.225
GO3	0.3	225	750	1500	7.5	0	0.375
GO4	0.3	225	750	1500	7.5	0	0.5625
GO5	0.3	225	750	1500	7.5	0	0.75

**Table 4:** Pore parameters of PCC, GO2, GO3, and GO5 mortars.

	Porosity/%	Average Pore Size/nm	>100 nm/%	<100 nm/%
PCC	8.0751	33.64	3.96	4.11
GO2	6.8643	24.88	1.93	4.93
GO3	6.9397	26.54	2.05	4.89
GO5	11.5223	41.11	6.94	4.58

## Data Availability

The original contributions presented in this study are included in the article. Further inquiries can be directed to the corresponding authors.
